# Second International Conference of Chief Editors of Research Journals organized by Islamic World Science Citation Center (ISC) (Shiraz, Iran December 1-2, 2014)

**DOI:** 10.12669/pjms.311.7094

**Published:** 2015

**Authors:** Shaukat Ali Jawaid

Shiraz (Islamic Republic of Iran): Islamic World Science Citation Center (ISC) organized the Second International Conference of Chief Editors of Research Journals at the ISC Campus here on December 1^st^ and 2^nd^ 2014. It was attended by Chief Editors and representatives from Turkey, Iraq, Syria, Pakistan, and Malaysia besides Iran. After the welcome address by **Dr. M.R. Falahati** from the Regional Information Center for Science and Technology (RICeST) **Dr. Razmjoo** Director General of Fars Governorship, Education and Research Office addressed the conference participants. He highlighted the importance of Shiraz the cultural capital of Iran which has a rich historical background. He mentioned the Shiraz University, famous poets like Hafez and Saadi besides Persepolis which attracts tourists to Shiraz. It is also famous for Center of Excellence for Science and Technology. Speaking about the Shiraz University of Medical Sciences, he said that it has 2, 50,000 students and ten thousand faculty members which includes 250 Professors, 300 Associate Professors. There are more than one hundred training centers in the Fars Province. In addition there are fourteen universities and Research Centers including SUMS, Arts, FASA, Jahrom University, Payam Noor University, College of Technology and Islamic Azad University. He also disclosed that the top five authors from Iran with maximum citations are also from Shiraz University. Shiraz University of Medical Sciences has a well established Liver Transplant programe.

Some of the strategies which the government, he said, has worked out were linking research centers with other institutions and organizations. We intend to establish knowledge based companies to produce wealth. We are also establishing Science and Technology Zone besides working on the scientific development of the province. We have plans for implementing comprehensive Map of Science, development of Applied Nano Technology and connecting university academicians with industry. We also plan to connect university research centers with Islamic countries overseas. He suggested that the participants should give suggestions to promote indexing of journals in ISC. We also need to penalize those who indulge in plagiarism as it involves various important ethical issues. He concluded his presentation by offering complete support of Fars Province to the ISC.

**Dr. Abbas Sadri**, Director of ISESCO Regional Office in his speech emphasized the importance of achieving self sufficiency in Science, Technology and Economy. Islamic countries, he opined, must evaluate Science and Technology and ISC can help these countries in this regard. ISC he further stated has an important role to play. Government, Policy makers, University Managers all needs reliable science and technology. Production of scientific literature and number of publications from different conies are some of the important indicators. He also emphasized the importance of quality journals, evaluation of individuals and institutions, Citation Database and who have cited these papers matters a lot. He commended the contributions of Prof. Jafar Mehrad and said that we are going to develop Science and Technology as we need quality research and quality information. He also highlighted the importance of Science Database, Citations, steps taken to promote Science and Technology. ISESCO, he said has organized over one hundred conferences and workshops this year. ISC has achieved international recognition and credibility within a span of just six years and it is an important asset for the Muslim World, he remarked.

Addressing the conference participants **Prof. Jafar Mehrad** President of ISC said that at present ISC was the third major database after ISI and Scopus which covers four thousand journals from OIC as well as non-OIC countries which include 1046 journals in Science and Technology. Journal Citation Report are published in English, Persian and Arabic while it plans to cover French language as well in the near future since French is spoken in many Muslim countries. This was stated by Prof. Jafar Mehrad President of Islamic World Science Citation Center while speaking in the inaugural session of the second International Conference of Editors of Science Journals held here at the ISC campus on December 1-2^nd^ 2014. It was attended by invited Editors of Science Journals and representatives from many Muslim countries including Malaysia, Pakistan, Turkey, Syria, Iraq and Islamic Republic of Iran.

ISC, Prof. Mehrad stated will remain at the cutting edge all the time. He also talked about historical background as to how the idea of ISC was conceived at a meeting of Ministers of Higher Education, Science and Research in Kuwait in 2006 which ultimately materialized at the 4^th^ conference at Baku in Azerbaijan in October 2008. Iran was given the responsibility to establish it. Assessment of Research, Prof. Jafar Mehrad said was a very complicated phenomenon. ISC, he further stated was a bit different from other two important databases i.e. ISI and Scoups since they both cover English literature and ignore other languages. Maintaining a database is a time taking process. While funding is not a problem, the hindrance is human resource training and development of software. He also disclosed that ISC has started an MS programme in Scientometrics to train human resource needed to develop software’s. ISC plans to establish branches in member ISESCO countries and it will require training of expertise.

ISI, Scopus and ISC, Prof .Jafar Mehrad further stated undertakes various types of assessments and rankings through a number of indicators including researchers, scientific journals, subject fields universities, research institutions and countries. Both ISI and Scopus are well known citations systems embodying a number of interesting products and services of which the scientific community can avail it. Despite this they fail to cover local languages but despite its short history, ISC has endeavored to cover languages other than English as well. Giving details of the journals at present covered by ISC he mentioned 1117 Arabic Journals, 1056 English, 403 from other languages which are indexed in ISC. The number of journals covered from Iran includes 1046 affiliated to the Ministry of Science, Research and Technology, 331 journals from Ministry of Health, Treatment and Medical Training while 250 Journals which are affiliated with the Islamic Azad University. 

ISC software, he said, has been developed by our own staff. We consistently improve and enrich it by adding new features and new products. At present twelve students are studying in MS in Scientometrics stated at RICeST and hopefully in the coming few years this will fill the gap of the required manpower. Establishment of ISC branches in Muslim countries will accelerate the enlargement of ISC database. However, it will require training of expertise. To improve the quality of journals, he suggested that Editor-in-Chiefs and Members of the Editorial Board should be picked up from outstanding medical personalities and researchers. The status of academic journals should also be enhanced through peer review process. Qualified and experienced reviewers can guarantee the quality of manuscripts and their originality, he remarked. Referencing mechanism used in journals also needs to be looked into as studies have revealed out-text citation standards. Inconsistencies have also been observed in journals even between different issues of the same journal and even at time within the different articles in the same journal.

Continuing Prof. Jafar Mehrad said that because of developments in e publishing, majority of the journals are easily accessible. Use of Online submission system by various journals website will help them develop their own archives. Plagiarism is yet another important issue which he opined needs to be taken seriously. He also proposed establishment of a Publishing Agency which should publish journals, get them indexed for which he assured financial assistance. Out of four thousand journals covered by ISC, about one thousand, he stated, have got an Impact Factor. ISC indexed journals should visit ISC regularly and ISC conferences should be organized in different countries for which it will provide financial assistance. He urged to promote and use ISC Citation, hold regular workshops at National, International level to publicize research to highlight scientific potential of the Muslim countries.

**Issues and Challenges**

The first scientific session was devoted to Issues and Challenges. **Mr. Shaukat Ali Jawaid** Managing Editor of Pakistan Journal of Medical Sciences was the first speaker who described his personal experience of how to find and retain good Reviewers. He suggested that all journals must maintain a Reviewers database of National, Regional as well as International Reviewers which should be constantly updated. The types of reviewers will depend on the scope of the journal. Those who have a poor track record of publications, those who maintain close relations with rival journals, those who have a reputation of being Nasty were mentioned as some of the potentials for exclusion.

Speaking about the selection criteria and qualifications of reviewers, he said that those selected must have adequate knowledge in their respective areas. There are some good reviewers, some not so good and Excellent reviewers. Editors must always review the reviewers comments, edit them if need be before sending them to the authors. Editors should have an author friendly and reviewer friendly policy. Excellent reviewers will also edit, correct English and Grammar besides carefully looking at the references. Sources of Reviewers include academic institutions, Centers of Excellence, professional specialty organizations, faculty members, speakers at conferences, authors besides different databases.

In order to retain good reviewers, always appreciate and recognize their services. A letter of Thanks after they review a manuscript is very helpful. However, one should refrain from overburdening the good reviewers. CME credits hours, elevating good reviewers to the Editorial Board of the Journal, providing them good reading material, books, meeting them from time to time, appreciation certificates, providing them opportunities for training at workshops, reduction in publication charges, post review thanks letter besides awarding the distinguished Reviewers were mentioned as some of the measures which can be taken. In case you can afford, financial rewards was yet another option for retaining good reviewers.

**Dr. Sholeh Aarstoo Poor** from the Regional Information Center for Science and Technology (RICeST) from Iran spoke about Review Articles: The art of taking mutual benefit through saving time. She pointed out that in Review one has to handle explosion of information like a professional. Review papers, she said, summarize the current literature on a topic. These Reviews have a very high readership and Editors appreciate good Reviews. They are also appreciated by the scientific community. It also leads to high Impact Factor for the journals because of increased citations that is why Annual Review Journals have a very high Impact Factor. Review Papers help readers, access current status of related field and they also act as link to other papers. They also help young scholars to interpret. Quality reviews require sifting and filtering the information. Narrative Reviews, she said, provide broad overview of specific topic while Systemic Reviews have narrow scope with specific question to be answered. Authors for such reviews are usually selected by the Editors keeping in view the expertise of the author.

Continuing Dr. Sholeh Aarastoo Poor said that review papers should be seen by variety of people. The literature base which has to be reviewed is important. It is also important to decide the literature search strategy. The Reviews must be update with latest literature as some databases are more reliable. Systemic reviews, she opined, are more fruitful than narrative reviews. Most of the time authors who are requested to write Reviews are given guidelines by the Chief Editor of the journal, she added.

**Dr. Mohammad Reza Ghane** from RICeST gave details about Open Access Policy. He pointed out that this initiative in scholarly communication was first taken by the Royal Society of London and French Academy of Science in mid 17^th^ century. This was triggered by the problems faced due to increased cost of publications, permission crisis, universities could not pay for increased cost of the journals while requests for copy rights was yet another problem because publishing companies used to make money as they always asked for the transfer of rights. The advent of internet made a great difference and new technology was combined with the traditional one. At present Dr. Ghane stated that 45.1% of internet users are in Asia, 20% in Europe, 10.7% in North America and 3.7% in Middle East. On the whole over three million people were currently using internet.

 In Open Access Publishing, it is the researchers, authors who pay for publication to ensure open access. The Gold Route is that the researchers publish their findings in open access journals. Some journals have a policy of delayed open access and the time period varies from six months to twelve or in some cases it may be two years. The Green Route means self archiving of research findings in repository. There are institutional repositories and subject based repositories. It was in Budapest in 2002 that finally Open Access initiative was taken. Bethesda supported open access in 2003 while Berlin Declaration on open access was also announced the same year in 2003. Dr.Reza Ghani was of the view that we must know about copy rights and respect it as well. He then threw light on attribution to share article, attribution by Non Commercial while NC-ND was the most restrictive.

Dr. Reza Ghani then showed a number of websites to share information. These included the Directory of Open Access Journals (DOAJ) which has over ten thousand journals from one hundred thirty six countries having 1.5 million manuscripts in its database. This covers almost one third of the world’s scholarly journals. The next website he showed was of Directory of Open Access Books (DOAB). It has 2426 peer reviewed books from seventy nine publishers. It has about 40% annual growth for books and publishers. Directory of Open Access Repository (DOAR) has lot of manuscripts from 2006-2014. BASE and High Wire were other important and useful databases. PubMed Central has large number of manuscripts starting from 2008 to 2014. Some other databases which were mentioned included OrXIV.org of Cornell University which has an 11% growth rate, DOARMAP- Repository Mandatory Archiving Policies, RePEC is an economic database. Yet another important database is of Social Sciences Research Network. ISC database which covers over four thousand journals provides open access and this in return ensures greater citations. Original articles are cited more frequently. Elsevier, Springer and Wiley’s have their own open access policies. However, the quality of open access journals, Dr. Reza Ghani opined was very important.

**Dr. M.R.Falahati** from RICeST Iran talked about the ISC Indexed Journals and their English Quality Assessment. He looked at the quality of English abstracts in Iranian journals. This study covered twenty four journals indexed by ISC in basic sciences during 2011-2013. One issue of each journal was taken. Pathology journal had 66 total errors; IAU Basic Sciences had total 167 errors. He pointed out that we have problem with quality of English and it also shows how much importance we give to Abstracts. In all he listed 1439 errors of English and Grammar. These errors consisted of space problem, punctuation, use of word “The”, use of lower and upper case of letters, spelling and use of redundant terms. In some cases general guidelines for authors were not adhered to and the length of manuscript was not as per the journal guidelines.

He then informed the participants about the availability of software APA in English and APA in Persian. Then he demonstrated errors in some other journals from OIC countries wherein the key words in the manuscripts were missing. We in the ISC, Dr. Falahati said are doing our best to provide infrastructure and databases.

**Prof. Sarinah Low** Binti Abdullah from University of Malaysia, Chief Editor of Asian Pacific Journal of Public Health emphasized the importance of international collaboration in scientific publishing. International collaboration, she stated was initiated in the last decade. International co-authorship publications are now increasing and it also ensures more citations. She was of the view that international collaboration also improves the quality of research. Journals published from this region, Prof. Low opined, vary in quality. There is great competition for quality papers and publication of journals has many problems. We can overcome some of these problems by pooling our resources, cutting cost of production and publications. She also laid emphasis on intellectual collaboration, training and management.

Continuing Prof. Low said that at present Turkey, Iran, Egypt, Jordan and Saudi Arabia has highest number of publications while Turkey enjoys the First position. Saudi Arabia and Jordan both have collaboration with international authors. Turkey and Iran, she said, are going forward through partnership with developed Nations. Both these countries are making advances in Science and Technology. Prof. Low then talked about collaboration in the Western Pacific Region. International collaboration, she further stated, was important to increase Impact Factor. We must understand the dynamics of international collaboration. While publication is the end result, we need good quality research. She laid emphasis on capacity building, training courses for Editors at Regional level, exchange of training material on Editing and Peer Review.

**Dr. Ali Akbari Sari**, Dean School of Public Health at Tehran University of Medical Sciences who is also Secretary of Commission of Medical Journals Auditing in Ministry of Health and Medical, Government of Iran made a presentation on Medical Journal publishing in Iran. He pointed out that the number of medical journals published from Iran has increased from 90 in 2004 to 345 in 2014. Hundred percent of these journals practice double blind peer review system. Some of them do receive some fee for fast track processing. Almost 95% of these journals, he said, have active functional websites and all these journals are indexed in ISC. Again 95% of these journals have Abstracts in English language and one hundred eighty six journals have full text in English language. Seventy five journals have XML full text. Each journal publishes about sixty papers every year and the total number of papers published from Iran annually was about twenty thousand.

Speaking about the problems faced by these journals, Dr.Ali Akbari Sari said that about 10% face delay in publication of one to two issues. Tehran University of Medical Sciences published fifty journals while the School of Public Health affiliated with TUMS alone publishes eleven journals. Majority of these journals, he said, are owned by medical universities in Iran. They have office in the respective university; journal staff is appointed and affiliated with these universities. Seventy two of these Iranian Journals, he said, are covered by PubMed, seventy eight by EMBASE and seventy eight by SCOPUS.

Talking about the challenges they face, he mentioned non availability of professionals, Diseconomies of small scale, Delay in peer review, delay in publishing, low citations, publication ethics, distribution of type of papers as some have just Reviews while others have original articles only. It is essential that all these journals should have a variety of contents. He also gave details pertaining to the working of the Journals Commission in the Iranian Ministry of Health, how they help and support these journals, accredit and Re-accredit these journals. These medical journals, Dr.Ali Akbari Sari remarked are also offered financial support, they are helped in indexing, training of their staff, preparing the journal websites and journal’s databases.

During the discussion Mr. Shaukat Ali Jawaid pointed out that too much importance was being given to Impact Factor which was just one of the criteria to judge the quality and standard of a journal. There are many other criteria’s and each one of them has its own value. Prof. Mehrad remarked that even while preparing the Journal Citation Report, different factors are taken into account for Ranking. I am a member of the Shiraz University of Medical Sciences which determines the promotion of faculty members and there too we take into account various criteria’s, he remarked. One of the participant opined that apart from the Impact Factor, the value and scope of research should also be looked into. Another participant suggested that while preparing ranking, the number of faculty members and the number of publications should also be compared. An institution having a very large faculty is likely to have more publications but if one work out number of publications per faculty members, it may be different. Hence it is essential that while looking at absolute statistical figures, relative statistics was also given due importance. It was also emphasized that contribution of the study should also get due importance after Impact Factor. Dr.Alizadeh referred to some of the weaknesses of Impact Factor wherein all citations are treated equally while the journal which cites these published manuscripts was also important. Hence, it is essential that we look at new approaches to look at the standard of journals and determine ranking of various authors, institutions. Replying to a question, it was stated that ISC products are freely available which was different from the practice by ISI Reuter/Thompson. ISI, Dr. Reza Ghani said also uses different classifications and names i.e. Q1, Q2, Q3 and Q4. Other participants suggested that we should hold training workshops for Peer Reviewers on regular basis.

**Understanding of ISC**

The scientific session on Day-2 was devoted to understanding of Islamic World Science Citation Center (ISC) and all the five presentations in this session were from the staff of ISC. **Ms. Forough Rahimi** was the first speaker who opined that ISC Journal Citation Report is one of the most important products of ISC. It provides quantitative tools for ranking, evaluation, categorization and comparing Islamic World’s leading journals. ISI, she said, was established in 1960, Scopus got established in 2004 while ISC made its debut in 2008. ISC covers journals from the Muslim World besides some other countries as well. ISC also helps measure the research impact. ISC (www.isc.gov.ir) also shows highest Impact Factor journals in a country. Measures of the Journal Citation Report (JCR) include Impact Factor, cited half life besides citing half life. She then demonstrated how the ISC website can be accessed and used. ISC database has at present 15,429 articles from Malaysia, UKM has contributed 2006 manuscripts and about 38% of articles covered from Malaysia were in Humanities. The number of articles recorded from Iraq was 32,505. By using the ISC website, one can also track the number of publications from different countries and institutions. ISC database also provides information about authors, institutions besides the number of papers published. Most articles covered from Iraq are also from Humanities followed by Health Sciences. She also disclosed that many Non-Muslim countries have also approached ISC to include their journals in this database.

**Dr. Hajar Safahieh** gave details about the ISC Journal Citation Report. This, it was stated, was one of the most important products of ISC. It provides quantitative tools for ranking, evaluating, categorizing and comparing Islamic World’s leading journals. It also helps measure research impact besides showing the Impact Factor of journals in a country. She then demonstrated how to access JCR on ISC website, how to use English Journal Citation Report (EJCR). Immediacy index, she said, is the average number of times an article is cited in the year it is published. She then gave details about different other information which is available on EJCR. This, it was stated, is a useful tool for evaluating, ranking of Islamic country journals, identifying highly cited journals in different fields. It is helpful for faculty, students, librarians, hospital administration and authors.

**Dr. Ali Ghazni** talked about ISC World Scientific Contribution Reports. He showed the ISC Master Journals list on the ISC website. ISC World’s scientific contributions report gives details of number of publications from different regions as well. North America, it was stated, has most of the publications. China had 40% of the papers from the region in 2013 which shows the tremendous amount of research work going on in China. During 2013, Pakistan had 2013 papers in the ISC while Turkey had 10,309 papers in ISC which accounts for 5% contribution to the world literature. Iran had 1.5% of world publications in 2013. It was also pointed out that there has been a rapid growth in the number of papers from Iran over the years. Core publications are a new concept developed by ISC. It also gives information published by different countries in top ranking journals and the details about their Impact Factor. Iranian Mathematicians had the most papers in top 1% of Journals while Pakistan Mathematicians had 72 papers in top 1% of Journals. On the whole in 2012, six hundred papers from the Muslim world were published in top 1% of Journals. Similarly 363 papers from low income countries were published in top 1% journals in Medicine. Most Middle East countries authors publish in low IF journals. One can compare information of different countries with OIC countries as regards their publications. Pakistan had a dismal figures compared with other OIC countries. One can use this source to analyze any Muslim country with other Muslim countries, other countries while it also gives information about the number of publications in a particular year.

**Dr. Hamid Alizadeh** discussed why we should evaluate the journals and who benefits from it? Authors benefit from the ranking while Librarians use it to subscribe good quality journals. Publishers and Editors use it to compare it with other journals. All citations, he pointed out, are not equal. The quality journals will attract good quality manuscripts. Impact of a single citation is given more importance in the relevant subject where citations are less likely. He then gave details about five journals. Pakistan Journal of Medical Sciences was the most productive in 2013 as compared to five other journals from Iran and Malaysia while Asia Pacific Journal of Public Health from Malaysia achieved the No.1 position overall.

**Dr. Bahareh Pahlavan Zadeh** in her presentation introduced the XML Journal submission system. It was found much more adoptable to various systems. This, she said, had many advantages i.e. less work load, ability to standardize the process. It provides comprehensive information. She then introduced ISC Journal Submission System and gave details of the compulsory information to be filled in this system. It helps authors, it is user friendly and Arabic interface will be developed soon, she remarked.

During the discussion, Dr.Falahati remarked that we are open to suggestions and constructive criticism. Prof. Mehrad remarked that many Non-Muslim countries like USA, UK, Poland, Germany, India, and France are interested to be covered by ISC. We have our own rules and regulations by Ministry of Science and Technology of Iran as regards Iranian Journals and the second is related to international journals. We are looking into these issues and hopefully it will be open to all journals. Responding to a question as to what was the motivation for these Non-Muslim countries to be covered in ISC, it was stated that this will ensure them more citations. All this will make it an international citation system and we will be glad and happy on this development. One of the participants asked about the criteria used to select the journals for ISC? Responding to this Prof.Mehrad said that we have evaluation criteria. Initially we indexed 370 journals from other countries which were not eligible; hence they were later removed from the list.

Responding to another question from Prof. Low, Prof.Mehrad said that we are not rivals to the ISI or SCOPUS. In fact we have close collaboration with both these important databases. We enjoy working together. They do their job and we do our job. We have commitments to the Islamic countries. Preparing Citations System requires some time. During the last six years ever since the inception of ISC in 2008, we have made tremendous progress. The whole Muslim World should be proud of it. Each system has its own criteria. We are not publishing journals but we are processing the journals. ISI and SCOPUS have their own systems of indexing and we have our own. Answering another question as to why we say ISC is the Third in ranking as regards different databases, DR. Falahati said that it is just based on the number of years they have been operating. ISI is the oldest followed by SCOPUS while ISC was established just six years ago. That is why we say it is the Third in Ranking, he remarked.

In his concluding remarks Prof. Jafar Mehrad President of ISC said that we are doing our best. Let us strengthen our ties in the field of Science and Technology in the Muslim countries. We must all pay attention to ISC and promote it. We need help and assistance of every one to promote and further strengthen this Database. Information available on the ISC can be used by everyone and we will be sharing the ISC products with all the countries, he remarked.

**Executive Committee Meeting**

An Executive Committee meeting of ISC was also held on Day-2 of the conference which was presided over by Prof. Jafar Mehrad President of ISC. In his introductory remarks Prof.Mehrad emphasized the importance of improved cooperation with ISC. This, he opined, does not require much work or funds. He asked all the EC members to think about ISC in their home country and introduce ISC. We will help you to come and visit Shiraz and ISC Center. We are planning to go to Turkey and other Muslim countries for similar meetings. We have divided Muslim countries in different regions. Malaysia has a representation in ISC; Pakistan can represent Middle East except Arab countries. We can have representation from Central Asia, Iraq and Syria can represent Middle East while Lebanon can represent Africa. We should provide a network for ISC Executive Committee members. The most important function of the Executive Committee members, Prof. Mehrad said, was to think about ISC and work for its further development and strengthening it further. We wish to have a branch in each Muslim country. We hope to establish a branch in Pakistan as well. Prof.Mehrad also disclosed that they have recently started an MS programme in Scientometrics at Shiraz University and Executive Committee members can help find eligible students for this programme. We wish to have a Focal person in each Muslim country. Publication of a Journal of Scientometrics which can be published twice a year was also discussed.

Mr.Shaukat Ali Jawaid Chief Editor of Professional Medical Publications publishers of fortnightly medical newspaper Pulse International and an international peer reviewed biomedical Journal “Pakistan Journal of Medical Sciences” said that he will contact the concerned authorities in Pakistan besides discussing this issue with University of Health Sciences at Lahore to set up an office of ISC in Pakistan. Another suggestion put forward during the discussion was of Discussion Group on ISC website. Prof. Jafar Mehrad said they will welcome research proposals from the Muslim countries researchers for financial assistance. ISC was also prepared to publish journals, books besides organizing training workshops for the Editors of Journals. Prof. Low from Malaysia also stressed the importance of capacity building workshops by training the editors.

**Figure:**
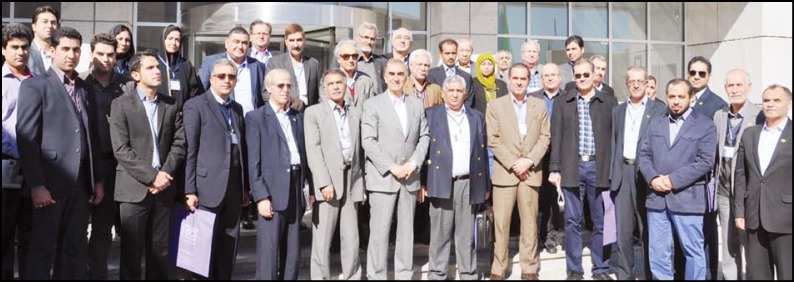
*Prof. Jafar Mehrad President Islamic Science Citation Center photographed with some of the foreign delegates at the Second Int. Conference of Editors of Scientific Journals org anized by ISC in Shiraz from December 1-2nd 2014.*

**Figure:**
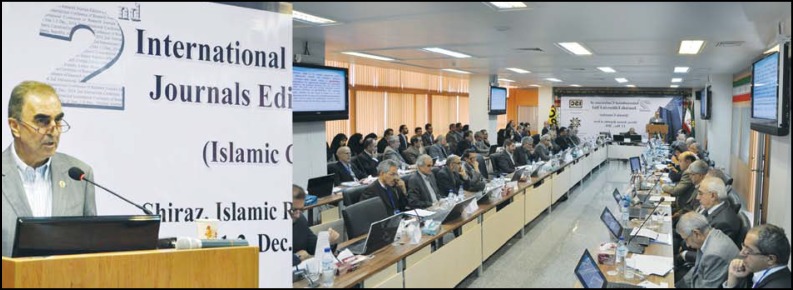
*Prof. Jafar Mehrad President ISC addressing the conference participants.*

**Figure:**
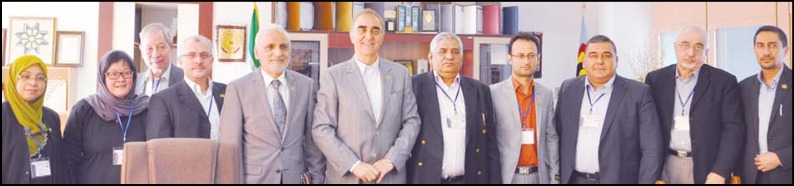
*ISC Executive Commitee Meeting was held on December 2nd, 2014. Photograph shows members of the Executive Commitee along with Prof. Jafar Mehrad President ISC after the Meeting.*

Responding to a question from Dr.Anita who represented Malaysian Citation Center, Prof. Jafar Mehrad said that different countries can have their own Citations Centers if they wish but we can work in close collaboration. Replying to yet another question Prof.Mehrad pointed out that all functions in ISC were similar to ISI. We have close collaboration with ISI. We are using their JCR and all other products especially the same software’s. We wish to have close collaboration of different citations centers in different countries with ISC. The work being done in various countries is very useful. ISI is working in close collaboration with China and Korea and both these countries have developed their own Databases. They could not have done it at their own without the help and assistance of ISI, Prof. Mehrad remarked.

